# Wire-Free Targeted Axillary Dissection: A Pooled Analysis of 1300+ Cases Post-Neoadjuvant Systemic Therapy in Node-Positive Early Breast Cancer

**DOI:** 10.3390/cancers16122172

**Published:** 2024-06-07

**Authors:** Jajini Varghese, Neill Patani, Umar Wazir, Shonnelly Novintan, Michael J. Michell, Anmol Malhotra, Kinan Mokbel, Kefah Mokbel

**Affiliations:** 1 The London Breast Institute, The Women’s Health Centre, HCA Healthcare UK, London W1U 9QP, UK; 2 Royal Free London NHS Trust, Pond Street, London NW3 2QG, UK; 3 Division of Surgery and Interventional Sciences, University College London, London WC1E 6BT, UK; 4 East Suffolk North Essex NHS Foundation Trust, Turner Road, Colchester CO4 5JL, UK; 5 Kings College Hospital NHS Foundation Trust, Denmark Hill, London SE5 9RS, UK; 6 Health and Care Profession Department, Faculty of Health and Life Sciences, University of Exeter Medical School, Exeter EX1 2HZ, UK

**Keywords:** breast cancer, node positive, systematic review, targeted axillary dissection

## Abstract

**Simple Summary:**

Preoperative chemotherapy significantly improves response rates in early breast cancer, challenging the traditional approach of invasive axillary surgery in patients presenting with node-positive disease. Wire-free localisation markers can successfully mark lymph nodes at diagnosis. Our findings demonstrate that these markers are successfully utilised, localised, and retrieved intraoperatively. Incorporating preoperative lymph node marking into the current biopsy procedure allows for more accurate staging of the axilla whilst reducing the need for invasive axillary surgery. These findings underscore the importance of incorporating both types of biopsies in axillary staging following preoperative chemotherapy for initially node-positive patients.

**Abstract:**

Recent advances in neoadjuvant systemic therapy (NST) have significantly improved pathologic complete response rates in early breast cancer, challenging the role of axillary lymph node dissection in nose-positive patients. Targeted axillary dissection (TAD) integrates marked lymph node biopsy (MLNB) and tracer-guided sentinel lymph node biopsy (SLNB). The introduction of new wire-free localisation markers (LMs) has streamlined TAD and increased its adoption. The primary endpoints include the successful localisation and retrieval rates of LMs. The secondary endpoints include the pathological complete response (pCR), SLNB, and MLNB concordance, as well as false-negative rates. Seventeen studies encompassing 1358 TAD procedures in 1355 met the inclusion criteria. The localisation and retrieval rate of LMs were 97% and 99%. A concordance rate of 67% (95% CI: 64–70) between SLNB and MLNB was demonstrated. Notably, 49 days (range: 0–272) was the average LM deployment time to surgery. pCR was observed in 46% (95% CI: 43–49) of cases, with no significant procedure-related complications. Omitting MLNB or SLNB would have under-staged the axilla in 15.2% or 5.4% (*p* = 0.0001) of cases, respectively. MLNB inclusion in axillary staging post-NST for initially node-positive patients is crucial. The radiation-free Savi Scout, with its minimal MRI artefacts, is the preferred technology for TAD.

## 1. Introduction

### 1.1. Evolution of TAD

Due to the significant risk of morbidity, the use of complete axillary lymph node dissection (ALND) has been replaced by the less invasive sentinel lymph node biopsy (SLNB) as the preferred method for evaluating axillary lymph nodes in breast cancer patients with clinically negative nodes undergoing primary surgery or after neoadjuvant systemic therapy (NST) [1,2]. Recent trials, such as AMAROS [3] and ACOSOG Z0011 [4], have shown that excluding ALND for patients with a positive SLNB does not affect their overall survival (OS). These findings have sparked discussions about expanding the application of less invasive axillary surgery to include patients with clinically positive lymph nodes (cN1) who respond well to NST. This approach has been underpinned by recent advancements in neoadjuvant systemic therapy (NST), which have significantly improved pathologic complete response rates (pCRs) in patients presenting with node-positive disease.

Nevertheless, investigations into SLNB for patients with biopsy-proven positive nodes after NST have unveiled variable false-negative and detection rates [5]. A recent meta-analysis, encompassing over 3000 patients with node-positive breast cancer, revealed a false-negative rate (FNR) of 13% for SLNB alone following NST, breaching the desired threshold of 10% [5]. The analysed studies also exhibited significant heterogeneity and inconsistent results. The inconsistency of FNRs associated with SLNB in this context has been ascribed to anatomical alterations, leading to modified lymphatic drainage, NST-induced fibrosis, fat necrosis, granulation tissue formation, or tumour-specific characteristics [6]. Traditional SLNB alone was considered inadequate for axillary staging after NST and oncologically unsafe because under-staging could adversely impact local control and lead to suboptimal adjuvant therapy decisions. Sentinel node-guided sampling, removing three or more SLNs was reported to reduce the FNR to 8%, whereas it was 22% when fewer than three SLNs were detected according to a recent meta-analysis [7].

As a result, the next step was to investigate the potential integration of postoperative pathological evaluation of the lymph node confirmed by pre-NST biopsy. Our previous study illustrated that combining marked lymph node biopsy (MLNB) with SLNB in this scenario can achieve a relatively low false-negative rate (FNR) of 5.18% (95% CI: 3.41–7.54) [8]. The combination of MLNB and SLNB after NST for biopsy-confirmed node-positive breast cancer is referred to as targeted axillary dissection (TAD). The TAD concept was first introduced by Caudle et al. in 2015 [9].

Various techniques, including carbon or black ink tattooing and the use of markers like stainless steel, titanium, or polyglycolic acid clips, have traditionally been used to mark the lymph node confirmed by biopsy under ultrasound guidance before NST initiation [8]. This marking process is typically followed by a secondary localisation procedure to aid in identifying and retrieving the tagged lymph node during axillary staging surgery.

### 1.2. Main Wire-Free Localisation Technologies

Five wire-free localisation methods have recently been introduced [8], which have applications to axillary node marking. One option is carbon suspension, known as tattooing, which can be visually detected during surgery. However, it lacks pre-incision target node identification, requiring longer incisions and wider dissections. Rather than providing direct guidance to the marked node and minimising axillary dissection, this approach merely provides visual confirmation of the marked node when encountered during surgical exploration. Furthermore, its inability to be detected by ultrasound compromises accuracy [8]. The other four direct guidance technologies, utilising a radioactive iodine seed, a magnetic seed, an electromagnetic reflector, or a radio-frequency identification (RFID) tag, are probe-detected [8] and are the main focus of this review. These were initially introduced for the localisation of non-palpable breast lesions, although their application has recently expanded to the marking and localisation of pathological lymph nodes.

The technique of radioactive iodine-125 seed localisation (RSL), introduced in 1999, is employed for marking, identifying, and retrieving biopsy-proven lymph nodes in patients undergoing NST. These seeds, measuring 4.8 mm × 0.8 mm, are composed of titanium and encapsulate ^125^I, a gamma-emitting radionuclide with a half-life of sixty days. They are meticulously stored under controlled conditions within the radionuclide laboratory of the nuclear medicine department. Transportation occurs within sterile containers or pre-loaded into 18-gauge spinal needles, outfitted with specific materials to prevent inadvertent deployment. By adjusting the energy mode, the standard handheld gamma probes utilised for SLN detection can localise both the ^125^I seed and radioactive lymph nodes. Following the excision of the seed-containing lymph node, an intraoperative specimen X-ray confirms successful retrieval. The proper disposal of the ^125^I seed aligns with radioactive safety protocols, with guidelines in the United States recommending removal within 5 to 7 days. The application of RSL in TAD, commonly known as the MARI (marking the axillary lymph node with radioactive iodine seeds) procedure, was introduced by Caudle et al. in 2015 [9].

Magseed^®^, introduced in 2016, utilises a non-radioactive marker packed with iron particles. Its detection is made possible by a Sentimag handheld magnetometer, which generates an alternating magnetic field to magnetise the iron briefly. The Sentimag probe then identifies the small magnetic signature produced by Magseed^®^ (Endomag, Cambridge, UK). With dimensions of 5 × 1 mm, Magseed^®^ can be inserted under mammography, ultrasound, or computed tomographic guidance using a preloaded sterile 18-gauge needle and can be detected within 4 cm depth from the skin surface [9,10]. Magseed^®^ has primarily been used for marking breast lesions and/or lymph nodes before surgery. During surgery, the probe locates the seed within the lesion, guiding the surgeon accurately to facilitate its retrieval for pathological assessment [10]. The probe requires regular intra-operative calibration facing away from metallic objects and is not compatible with standard metal instruments. Plastic retractors and instruments are available for most surgical applications.

The SAVI SCOUT^®^ localisation system, introduced in 2016 and also known as radar reflector localisation (RRL) (Merit Medical in Aliso Viejo, CA, USA), offers a radiation-free approach with high clinical efficacy and exceptional accuracy, especially in reducing magnetic resonance imaging (MRI) artefacts. This has particular relevance for breast cancer patients receiving NST, where MRI remains the gold standard for monitoring treatment response and planning surgery. This approach involves the placement of a 12 × 1.6 mm electromagnetic wave reflector under ultrasound guidance, utilising a sterile 16-gauge introducer needle system. In the operating room, the reflector is activated using infrared light from the console probe, which reflects an electromagnetic wave signal back to the detection probe. This ongoing feedback assists in directing the surgical excision depth, enabling accurate depth detection of up to 6 cm from the skin surface [11]. The technology is compatible with all commonly used surgical instruments and does not require intra-operative calibration. However, the reflector is vulnerable to deactivation by direct contact with electrocautery devices, which should be used cautiously in proximity. 

The LOCalizer™ system, introduced by Hologic Inc. (Santa Carla, CA, USA) in 2019, is a wire- and radiation-free system that utilises an RFID tag (11 × 2 mm) and a handheld reader that displays the distance from the surface of the tag to the surface of the probe. The tag can be deployed under mammographic or ultrasound guidance using a preloaded 12-gauge introducer. Each RFID includes a unique identification number displayed by the reader [12]. This technology can therefore reliably distinguish between multiple tags intra-operatively. Unlike Savi Scout and RSL, where MRI artefacts measure less than 5 mm, magnetic seeds and RFID tags can generate signal void artefacts, potentially affecting the diagnostic precision of breast MRI while the marker is in situ. The MRI phantom measures approximately 2 cm for the RFID tag and 4 cm for Magseed^®^, yet ongoing technical advancements strive to mitigate the signal void artefacts associated with these technologies during MRI scans [9,10,11,12].

This systematic review and pooled analysis aim to assess the clinical performance of RSL, Magseed^®^, Savi Scout, and LOCalizer during TAD. The key outcome measures include evaluating successful localisation and retrieval rates, the concordance between MLNB and SLNB, and the incidence of pCR in clinically node-positive patients undergoing NST.

## 2. Materials and Methods

### 2.1. Literature Search

This study was approved by the multidisciplinary breast cancer board of the London Breast Institute. The literature review involved a comprehensive search of the PubMed and MEDLINE databases, including the National Library of Medicine, covering all relevant studies up to April 2024. This extensive search ensured that the most current and pertinent research was included in the review. The following keywords were used: [SAVI SCOUT] or [radar reflector localisation] or [Magseed] or [magnetic seed] or [radioactive iodine seed] or [MARI] or [LOCalizer] or [radio frequency identification tag] and [targeted axillary dissection] or [TAD] and [breast cancer] and [neoadjuvant]. Furthermore, additional studies were identified by searching bibliographies for potential inclusion, and the corresponding authors of relevant publications were contacted to address specific aspects of their data when needed. The systematic review adhered to the guidelines outlined in the Preferred Reporting Items for Systematic Reviews and Meta-Analyses (PRISMA). It should be noted that the protocol for this review has not been registered.

### 2.2. Selection Criteria

The studies identified in the literature review were evaluated based on the inclusion and exclusion criteria outlined below.

#### 2.2.1. Inclusion Criteria

Studies were considered for inclusion if they satisfied the following criteria:

- Single-centre or multicentre studies with a retrospective or prospective design;
- All patients had both SLNB and MLNB;
- Studies evaluating the role of RSL, magnetic seeds, radioactive iodine seeds, and RFID in TAD where patients underwent NST;
- The following primary data endpoints are available: successful localisation and retrieval rates of the LM;
- SLNB-MLNB concordance rate, pCR, migration rate, number of retrieved lymph nodes, and the duration between deployment and surgery were included in the analysis if available.

#### 2.2.2. Exclusion Criteria

The following criteria were used to exclude studies:

- Manuscripts not written in English;
- Studies involving non-human subjects;
- Non-peer-reviewed studies;
- Studies with 10 or fewer eligible cases;
- Conference reports and published abstracts only.

### 2.3. Statistical Analysis

For evaluating heterogeneity and creating a forest plot of successful localisation endpoints, we employed ‘STATA’ (STATA Corporation, College Station, TX, USA). GraphPad software (version 2024) was utilised to determine 95% confidence intervals and perform the chi-square test. Statistical significance was considered when *p* < 0.05.

## 3. Results

### 3.1. Literature Search Results

The initial search yielded 385 articles, with 21 appearing relevant [13,14,15,16,17,18,19,20,21,22,23,24,25,26,27,28,29,30,31,32,33] (Supplementary Figure S1). Further inspection narrowed down 17 articles meeting the inclusion criteria, covering 1358 procedures in 1355 patients [13,14,15,16,17,18,19,20,21,22,23,24,25,26,27,28,29] (Table 1). Three studies on 5 [30] and 10 patients were excluded [31,32], along with a fourth study on the feasibility and accuracy of RRL–MLNB in 86 patients, as all patients had ALND with no SLNB data [33]. Twelve of the studies that fulfilled the eligibility criteria were retrospective [1,13,17,20,21,22,23,24,25,26,28,29] and five were prospective [14,15,16,19,27], two of which were multicentre [14,27].

### 3.2. Pooled Analysis

**Figure 1:**
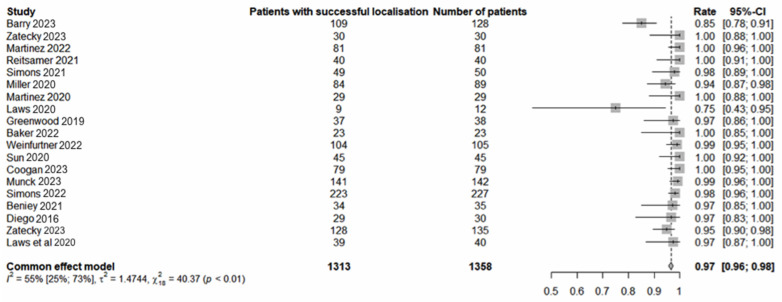
Successful localisation of localisation markers [13,14,15,16,17,18,19,20,21,22,23,24,25,26,27,28,29].

**Figure 2:**
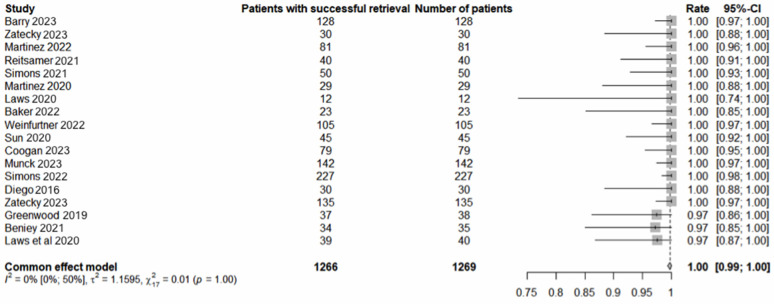
Successful retrieval of localisation markers [13,14,15,16,17,19,20,21,22,23,24,25,26,27,29].

**Figure 3:**
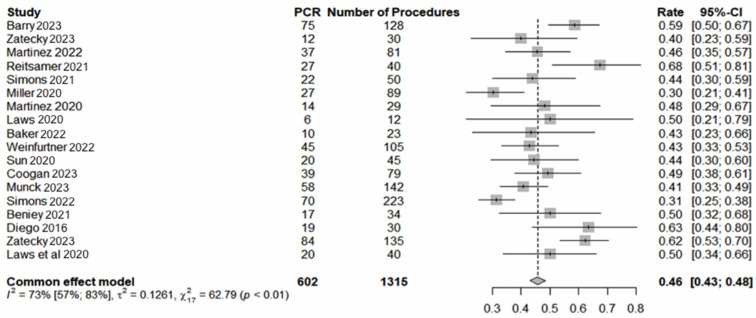
Pathological complete response [13,14,15,16,17,19,20,22,23,24,25,26,27,29].

Seventeen studies involving 1358 procedures in 1355 patients met the inclusion criteria. The pooled average age was 54 years (range: 22–92). The pooled analysis revealed a 97% (1310/1355) [95% confidence interval (CI) 96–98] successful localisation rate (Figure 1) and 99% (1350/1355) [95% CI: 99–100] LM retrieval rate (Figure 2). pCR was observed in 46% (602/1315) [95% CI 43–49] of cases (Figure 3). The average interval between deployment of the LM and surgery was 49 days (range: 0–272). The results, where available, are presented in Table 1. The pooled average number of lymph nodes retrieved was 2.3 (range: 1–11). The concordance between the MLNB and SLNB was observed in 712 of 1059 cases (67%; 95% CI: 64–70). Omitting MLNB or SLNB from TAD would have under-staged the axilla in 15.2% (66/434) [95% CI: 12–18.9] or 5.4 (27/502) [95% CI: 3.6–7.7] of cases, respectively. Compared with the final surgical pathology of TAD, the FNR of the MLNB-based axillary staging was significantly lower than SLNB-based staging (5.4% versus 15.2%; chi-squared equals 20.494 with one degree of freedom, two-tailed *p* < 0.0001).

## 4. Discussion

### 4.1. The Pooled Analysis 

In the pooled analysis of 17 studies comprising data on 1358 TAD procedures, excellent performance was observed across all four commercially available wire-free localisation technologies. The pooled 97% successful localisation rate and 99% successful retrieval rate of the localisation markers confirm the reliability of these technologies for TAD. Most of the data were derived from RSL (n = 574) and Magseed^®^ (n = 497) studies, followed by Savi Scout (n = 252). The use of LOCalizer was only reported in 40 cases [29]. The limited use of this technology may be attributed to the wide bore (12-gauge) of the introducer needle that could pose technical challenges to the deployment of the marker within mobile axillary lymph nodes [12]. Localisation failure was observed in 45 cases (29 with Magseed, 14 with RSL, 1 with Savi Scout, and 1 with LOCalizer). Most of the localisation issues with Magseed^®^ occurred during the secondary localisation procedure after completing NST [13]. Barry et al. found that Magseed^®^ was actually located in the adjacent axillary fat in 24.3% of cases when deployed after NST, compared with 1.8% when deployed before NST, emphasising the significance of single-stage localisation at the time of biopsy [13]. The identification failure of SLNs is another important factor contributing to TAD failure [14]. Failure to retrieve the localisation marker was only observed in less than 1% of cases (six with Magseed-TAD, one with RSL-TAD, and one with RFID-TAD). Marker dislodgement, a potential factor contributing to retrieval failure, was observed in 2.4% of cases in the IMTAD study, a prospective multicentre trial [14].

The average interval between deploying the localisation marker and surgery was 49 days (range: 0–272). The shortest duration was for RSL, mainly due to regulatory constraints in some jurisdictions. Nonetheless, the average interval of 49 days aligns with the flexible scheduling offered by wire-free technology. Given that the average duration of NST is between 90 and 180 days, the observed durations suggest that many localisation procedures occurred after completing NST. The pathological complete response (pCR) of axillary nodal disease was achieved in 48% of cases, predominantly associated with triple-negative breast cancer (TNBC) and HER2-positive disease, underscoring the efficacy of monoclonal antibodies targeting checkpoint and HER2 proteins [34]. In a recent meta-analysis of 33 studies, axillary pCR rates were 60% for HER2-positive/hormone receptor (HR)-negative, 48% for TNBC, 45% for HR-positive/HER2-positive, 35% for luminal B, 18% for HR-positive/HER2-negative, and 13% for luminal A breast cancer [34]. Consequently, patients with HR-negative/HER2-positive breast cancer are most likely to achieve axillary pCR and benefit from axillary surgery de-escalation, while those with luminal type A are least likely to achieve pCR.

The marked lymph node was also confirmed as an SLN in 67% of cases, with the FNR of SLNB being significantly higher than MLNB. The pooled analysis demonstrates that relying upon SLNB alone to stage the initially positive axilla after NST could potentially under-stage the disease in 15% of patients. This has potential implications for under-treatment with adjuvant regional nodal radiation therapy and subtype-specific targeted systemic treatment, which could adversely impact overall survival. The importance of including the MLNB in axillary staging post-NST to prevent under-estimating axillary involvement and subsequent under-treatment was reflected in the updated National Comprehensive Cancer Network (NCCN) guidelines for 2022. Hence, at least three lymph nodes should be harvested if sentinel lymph SLNB alone is employed to stage the axilla after NST [6].

The average number of lymph nodes harvested during TAD in the pooled analysis was 2.3 (range 1–11). The higher end of this range appears to be primarily influenced by the SLNB procedure, given that MLNB typically removes only 1–2 nodes. While removing additional lymph nodes and the total number of positive lymph nodes are often cited as risk factors for breast cancer-related lymphedema (BCRL), they are undoubtedly linked to the extent of surgical dissection, which likely represents the key driver of morbidity [35]. Given the relatively low FNR for MLNB (5.4%), it would be prudent to limit the number of sentinel lymph nodes harvested without significantly affecting the risk of axillary under staging. The enhanced prediction of axillary pCR is crucial for guiding the selection of breast cancer patients for TAD after NST in node-positive breast cancer. A consensus is emerging that multimodal predictive algorithms that incorporate nodal stage, ultrasound findings, MRI response, and molecular receptor status exhibit robust diagnostic performance in detecting residual axillary lymph node metastasis after NST in clinically node-positive patients [36]. However, it should be noted that the analysed studies had heterogeneous protocols regarding surveillance, criteria for response, and TAD eligibility. Additionally, many of the studies lacked these details.

This review demonstrates that all four localisation technologies performed well during TAD, with excellent successful localisation and retrieval rates, and no procedure-related complications. Each localisation technique has its own advantages and drawbacks, and there remains a lack of adequate studies comparing these technologies directly. The complex regulatory requirements represent a significant disadvantage for the radioactive iodine seed, particularly for facilities without a nuclear medicine department. Since contrast-enhanced MRI is the preferred modality for monitoring response to NST, localisation with Savi Scout has significant advantages over Magseed^®^ and LOCalizer, due to the generation of the smallest MRI signal voids. The minimal MRI artefact allows for single-step localisation with Savi Scout at diagnosis, which is time- and resource-efficient, in addition to improving patient experience and being more accurate than two-step localisation [13]. Another advantage of Savi Scout over the other two technologies is the greater depth of accurate detection [9,10,11,12].

### 4.2. Oncological Safety 

Since the introduction of TAD in 2015 [30], the use of this approach for staging the initially positive axilla post-NST has expanded rapidly. This growth is supported by increasing evidence of oncological safety and a shift in emphasis towards overall survival and quality of life as important endpoints in assessing the efficacy of breast cancer treatments. Nijveldt et al. reported that the introduction of the TAD procedure resulted in an 84% reduction in previously indicated ALNDs [37]. Additionally, 18% of cases did not receive adjuvant axillary radiotherapy. These data indicate that the de-escalation of axillary treatment with the TAD procedure has been successful. In another study, TAD assisted by 125I seed localisation of lymph nodes avoided ALND in 80% of cN+ patients, with a three-year axillary recurrence-free interval (ARFI) of 98% [38]. The five-year data from NSABP B-51 confirm the safety of axillary treatment de-escalation. The NRG Oncology/NSABP B-51/RTOG 1304 study supports omitting RNI in patients transitioning from cN1 to ypN0 status based on SLNB after NST [39].

For patients with positive axillary staging after NST, clinical trials are currently in progress to determine the optimal treatment strategy for the axilla. The ALLIANCE-A011202, a randomised Phase III trial, aims to assess whether radiation to the undissected axilla and regional lymph nodes can be as effective as ALND with radiation to regional lymph nodes, excluding the dissected axilla, in terms of recurrence-free interval for patients with positive SLNs after NST. Furthermore, two registry-based trials, AXSANA (NCT04373655) [40] and MINIMAX (NCT04486495) [41], both include patients regardless of their axillary complete response after NST. Finally, the MARI trial includes patients with clinically involved axillary lymph nodes (cALNs) <4 and positive ypMARI lymph nodes undergoing adjuvant axillary radiotherapy, as well as patients with cALNs ≥4 and positive ypMARI receiving ALND. The reported 3-year axillary recurrence-free interval was 98.2%. However, the five reported axillary recurrences all occurred in patients with cALNs <4, with four of these occurring in ypMARI-positive patients (resulting in a 3-year axillary recurrence rate of 3.4%) [42].

In patients with luminal-like breast cancer who achieve axillary pCR, Barbieri et al. reported no significant difference in OS or DFS between those undergoing ALND or SLNB only (*p* = 0.661 and *p* = 0.856, respectively) [43].

### 4.3. Limitations 

The current study represents the first comprehensive analysis of the performance of wire-free technologies during TAD, involving over 1300 patients. The findings highlight variable approaches in the analysed studies, including marker deployment timing, NST response monitoring, TAD selection criteria, and lymph node retrieval. Several studies were retrospective, with small sample sizes of under 100 patients, lacking direct technology comparison or oncological outcome data. Only two studies had a prospective multicentre design [14,27], one of which included a direct comparison between two technologies [14]. Moreover, the current analysis does not evaluate TAD performance in breast cancer patients with high initial lymph node involvement (≥3 clinically suspicious lymph nodes) due to data scarcity. Assessing the FNR of TAD versus ALND in ≥3 clinically positive lymph nodes in a larger cohort is crucial, given the potentially increased risk of false-negative TAD results with extensive initial lymph node involvement, in addition to the potentially greater adverse consequences of under-treating this higher risk cohort of patients.

## 5. Conclusions

All four wire-free and tattoo-free localisation technologies are deemed safe and reliable for facilitating TAD in initially node-positive patients who respond well to NST. The sole utilisation of SLNB seems notably less effective than TAD in accurately staging the axilla post-NST. TAD’s precise identification of axillary pCR enables the de-escalation of axillary surgery and regional nodal irradiation in nearly half of all breast cancer patients with low-burden axillary disease. Currently, the radiation-free Savi Scout stands out as the preferred wire-free technology for TAD, offering minimal magnetic resonance imaging artefacts, single-step localisation, and superior depth of detection.

## Figures and Tables

**Table 1:** Pooled analysis of included studies.

Study	Method	Number of TAD Procedures	Mean Age (Years)	pCR	Retrieval Rate	Localisation Rate (%)	Migration Rate (%)	Mean Implantation (Range) (Days)	Median Number of Nodes	SLNBL MLNB Concordance	FNR of MLNB	FNR of SLNB
Barry et al. [13]	Magnetic	128	59	59%	100%	85%	NR	20 (89–188)	2	59%	9%	23%
Zatecky et al. [14]	Magnetic	30	49	40%	100%	100%	0%	138.5	3.5	83%	0%	NR
Martinez et al. [15]	Magnetic	81	47	46%	100%	100%	0%	NR	1	81%	0%	11%
Reitsamer et al. [16]	Magnetic	40	52	68%	100%	100%	0%	NR	2.3	65%	0%	15%
Simons et al. [17]	Magnetic	50	NR	68%	100%	98%	0%	0–30	1.3	80%	NR	NR
Miller et al. [18]	Magnetic	89	58	30%	100%	94%	0%	NR	NR	NR	NR	NR
Martinez et al. [19]	Magnetic	29	55	48%	100%	100%	0%	10 (1–26)	1.2	50%	7%	21%
Laws [20]	Magnetic	12	51	50%	100%	75%	0%	(0–22)	3	NR	NR	NR
Greenwood et al. [21]	Magnetic	38	56	NR	100%	97%	0%	5 (0–31)	NR	NR	NR	NR
Baker et al. [22]	RRL	23	49	43%	100%	100%	0%	141	4	96%	NR	NR
Weinfurtner et al. [23]	RRL	105	57	43%	100%	99%	0%	35	NR	83%	NR	5%
Sun et al. [24]	RRL	45	55	44%	100%	100%	0%	8	3.5	80%	4%	NR
Coogan et al. [25]	RRL	79	51	49%	100%	100%	0%	80	3	68%	10%	28%
Munck et al. [26]	RSL	142	51	41%	100%	99%	0%	146.5	2	72%	7%	21%
Simons et al. [27]	RSL	227	52	31%	100%	98%	0%	NR	2	71%	6%	17%
Beniey et al. [28]	RSL	35	49	50%	97%	97%	3%	0	NR	NR	NR	NR
Diego et al. [29]	RSL	30	55	63%	100%	97%	0%	0	4	73%	0%	NR
Zatecky et al. [14]	RSL	135	51	62%	100%	95%	3%	1	3.2	27%	0%	NR
Laws et al. [20]	RFID	40	54	50%	98%	98%	0%	54 (0–272)	3	NR	NR	NR

NR—not reported.

## Data Availability

The datasets generated in this study are publicly available in this open access publication without any restrictions.

## Supplementary Materials

The following supporting information can be downloaded at: https://www.mdpi.com/article/10.3390/cancers16122172/s1, Figure S1: PRISMA flow diagram illustrating the inclusion and exclusion of studies reviewed for the analysis.
