# Supplementary material for: Wire-Free Targeted Axillary Dissection: A Pooled Analysis of 1300+ Cases Post-Neoadjuvant Systemic Therapy in Node-Positive Early Breast Cancer
Source: Cancers (Basel). 2024 Jun 7;16(12):2172. doi: 10.3390/cancers16122172 (PMC11201777; doi:10.3390/cancers16122172)
Supplement: Supplementary file 1 [file cancers-16-02172-s001.zip › cancers-3042284-supplementary.pdf]

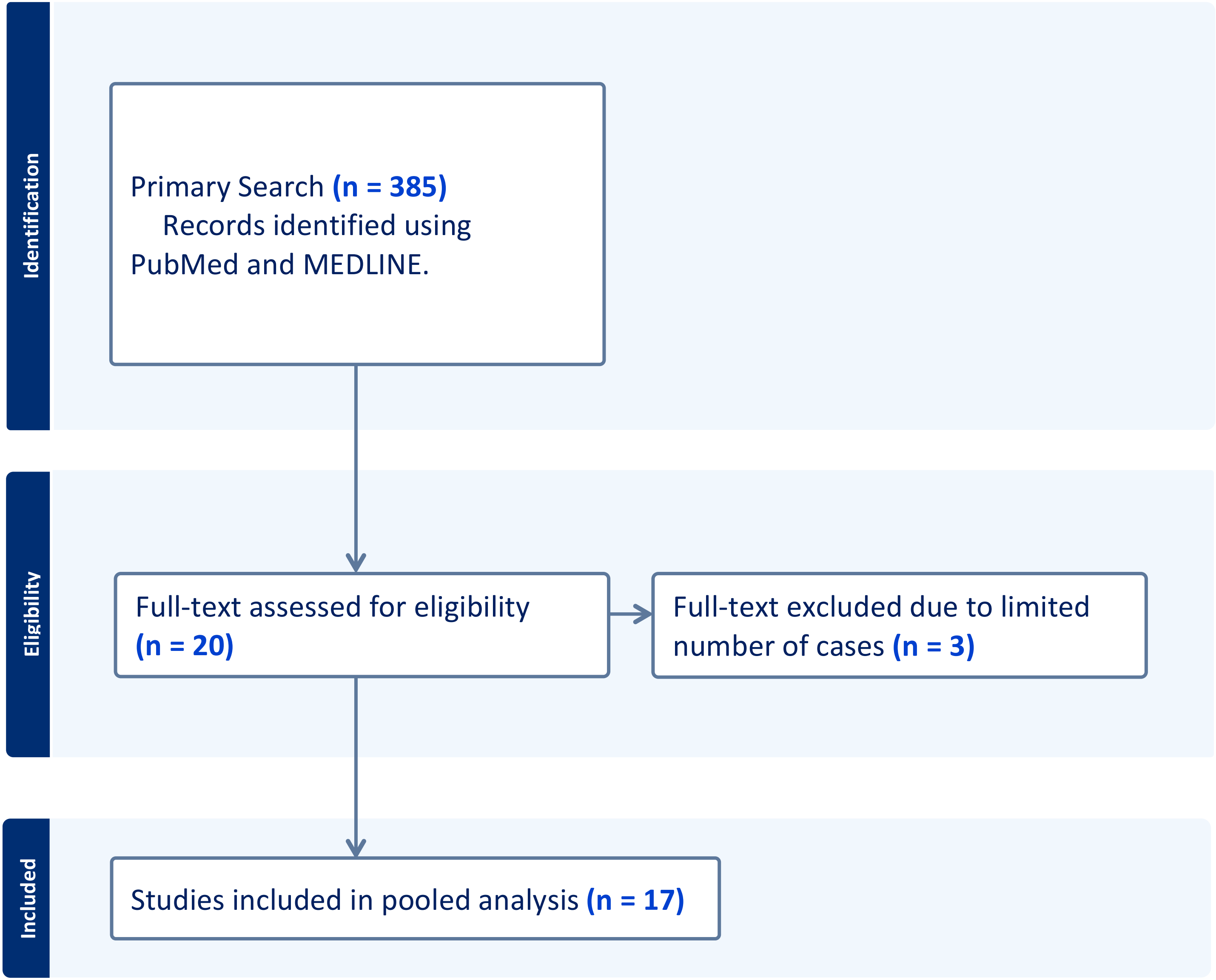

Figure S1. PRISMA flow diagram illustrating the inclusion and exclusion of studies reviewed for the analysis.
